# Coordination Dynamics in Cognitive Neuroscience

**DOI:** 10.3389/fnins.2016.00397

**Published:** 2016-09-15

**Authors:** Steven L. Bressler, J. A. Scott Kelso

**Affiliations:** ^1^Center for Complex Systems and Brain Sciences, Florida Atlantic UniversityBoca Raton, FL, USA; ^2^Intelligent Systems Research Centre, Ulster UniversityDerry/Londonderry, Northern Ireland

**Keywords:** cerebral cortex, interareal interaction, neuronal communication, event-related potential, local field potential, relative coordination, HKB model, computational context

## Abstract

Many researchers and clinicians in cognitive neuroscience hold to a modular view of cognitive function in which the cerebral cortex operates by the activation of areas with circumscribed elementary cognitive functions. Yet an ongoing paradigm shift to a dynamic network perspective is underway. This new viewpoint treats cortical function as arising from the coordination dynamics within and between cortical regions. Cortical coordination dynamics arises due to the unidirectional influences imposed on a cortical area by inputs from other areas that project to it, combined with the projection reciprocity that characterizes cortical connectivity and gives rise to reentrant processing. As a result, cortical dynamics exhibits both segregative and integrative tendencies and gives rise to both cooperative and competitive relations within and between cortical areas that are hypothesized to underlie the emergence of cognition in brains.

A shift to the dynamic network perspective is underway in cognitive neuroscience (Bressler and Kelso, 2001). Although neuroscience has made spectacular progress in the information-processing analysis of feedforward processes and input-output relations in the peripheral and central nervous system, it has lagged in understanding the well-documented reciprocal coupling of cortical neuronal structures. In the cerebral cortex, different areas are typically connected by unidirectional axonal pathways, with a projection in one direction reciprocated by a projection in the other direction. This reciprocal coupling provides new insights in cognitive neuroscience (Kelso et al., 2013; Razi and Friston, 2016), propelling a complex systems approach to the fore and playing a key role in understanding. Reciprocal coupling also proves to be essential in the fields of systems, cognitive, and social neuroscience. Feedback coupling among the component parts of a complex system supports the coordination of those parts and the emergence of a wide variety of nonlinear system behaviors (Bressler and Kelso, 2001). When coordination changes over time, the formation and dissolution of large-scale collective functional entities in the brain becomes possible (Kelso, 2014). Such large-scale, context-sensitive collectives underlying cognitive function are variously called neurocognitive networks (Bressler, 2008; Meehan and Bressler, 2012) or coordinative structures (Kelso, 1995, 2014; Kelso et al., 2013).

A paradigm shift appears underway in cognitive neuroscience that mirrors the emerging science of coordination dynamics (Kelso, 1992; Kelso and Haken, 1995; Tognoli and Kelso, 2009). Coordination dynamics provides a unifying framework for understanding the neurophysiological mechanisms underlying the integration and segregation of cortical areas in large-scale networks. A goal of coordination dynamics is to identify the key variables of coordination (defined as a functional and/or task-dependent ordering among context-sensitive interacting components) and their dynamics (rules that govern the stability and change of coordination patterns), and the nonlinear coupling among components that gives rise to them (Kelso, 1995, 2012; Kelso et al., 2013). In the context of cognitive neuroscience, the aim of coordination dynamics is to understand the functional interactions within and between different areas of the brain in relation to cognitive task performance (Bressler and Kelso, 2001).

Our approach is to identify neural components that are involved in a cognitive task, and to investigate how they change their relationships during task execution. The architectonically-defined area is taken to be the appropriate cortical component because long-range (white matter) axonal tracts are organized at the level of areas. The functional organization of the cortical area encompasses the interactions of neurons and glia in the local microcircuitry and neuronal populations in the local mesocircuitry. Cortical areas are treated as collections of locally interacting neuronal populations receiving inputs from, and sending outputs to, other areas. Of course, the internal dynamics of each cortical area influences the way it interacts with other areas (Bressler, 1995). Although we are sympathetic to the idea that neuronal communication operates in the cortex by means of principles of coherence (Singer, 1994; Fries, 2005), we emphasize that the concept of “communication” implies that one area acts as a sender that transmits a message to another area acting as a receiver, and that the receiver does not additionally act as a sender. Such single-direction interaction is very rare in the cortex, if it occurs at all (Bressler, 1987a,b). In general, like the component processes involved in behavior itself (Kelso et al., 2009, 2014; Dumas et al., 2014), cortical areas interact with each other reciprocally.

We consider the issue of how interareal cortical interactions lead to the emergence of cognition in real time. To speak to this aim, we investigate the large-scale cortical coordination dynamics that underlies the dynamics of cognition. As in any complex system, cortical coordination dynamics depends on the changing interdependency of cortical areas. Approaching the question of cortical interactions in this way allows us to utilize general coordination principles from the science of self-organization and pattern formation in open, nonequilibrium systems. These principles bring to bear the language and tools of coupled nonlinear dynamical systems for describing how cortical coordination patterns are initiated, and then persist, adapt and evolve in time (Schoner and Kelso, 1988; Kelso, 1992, 1995; Haken, 1996).

Central to the science of self-organizing coordination dynamics is the order parameter, or “collective variable,” that may uniquely define the relationship among a dynamical system's interacting components. In the brain, coordination dynamics displays both functional and context specificity. At the level of large-scale function, interareal relative phase (i.e., between neuronal populations in different cortical areas) is a crucial collective variable because relative phase dynamics captures the coordination among cortical areas. We emphasize that a number of different experiments using a variety of imaging modalities have demonstrated that relative phase in the cortex persists over time and then changes abruptly at state transitions (Fuchs et al., 1992, 2000; Kelso et al., 1992; Wallenstein et al., 1995; Mayville et al., 1999; Meyer-Lindenberg et al., 2002; Jantzen et al., 2009). This and related phenomena have led to proposals that the cortex undergoes characteristic sequential neurocognitive states as specific behaviors unfold (Freeman, 2006; Rabinovich et al., 2012).

Complex cognitive functions are known to be globally organized in the brain, but to also arise from elemental functions that are locally organized (Luria, 1980). Cortical function reflects both these global and local organizational aspects. What is beginning to be appreciated is that this duality fundamentally derives from the cortex functioning as a complex system with metastable coordination dynamics. The dynamics of metastable systems is characterized by both integrating and segregating tendencies acting in a highly complex, but balanced, interplay (Kelso, 1992, 2000; Tononi et al., 1994; Friston, 1997; Bullmore and Sporns, 2009; Deco et al., 2011; Tognoli and Kelso, 2014). Metastability is able to account for phenomena in the brain that have been described using ideas on self-organized instability (Solé et al., 2002; Friston et al., 2012), chaotic itinerancy (Breakspear, 2001), self-organized criticality (Shew et al., 2011), and multistability (Braun and Mattia, 2010); see also (Shanahan, 2010). Furthermore, metastability predicts the winnerless competition described in (Rabinovich et al., 2012).

The simplest, most pared-down mathematical description of coordination dynamics is a theoretical model of coordinative interaction in a (nonlinearly) coupled system of (nonlinear) oscillators. Called the HKB model, it was originally introduced as a theoretical explanation of: (1) the formation of ordered states of bimanual coordination between rhythmically moving limbs (treated as nonlinear oscillators); (2) the multistability of those states; and (3) the switching among coordinative states shown to be due to symmetry breaking instability (Kelso, 1995). The original HKB model described fundamental features of self-organization such as multistability, phase transitions, and hysteresis common to behavioral and neural systems (Haken et al., 1985; Schoner and Kelso, 1988). The model was later extended to include a noise term representing stochastic fluctuations (Schoner et al., 1986) and frequency differences between the interacting components (Kelso et al., 1990). This extended HKB model embodies a law of coordination that applies to many systems and may be said to be universal, hence independent of the specific structure of any particular system. The condition of special interest for cortical dynamics, called “broken symmetry,” occurs when the oscillatory components have different intrinsic frequencies (Kelso et al., 1990). This condition is crucial for producing the metastable dynamics that arises when cortical oscillators interact with each other (Kelso, 1992; Kelso and Haken, 1995; Bhowmik and Shanahan, 2013; Tognoli and Kelso, 2014). The HKB model extension (Kelso et al., 1990; Kelso, 1992, 1995) shows how the balance between interdependence and independence produces the relative coordination that is characteristic of normal cortical function. Relative coordination—so named by the behavioral physiologist von Holst (1939/1973)—provides cortical function with a flexibility that enables it to adapt to cognition's changing contingencies (Figure 1).

**Figure 1:**
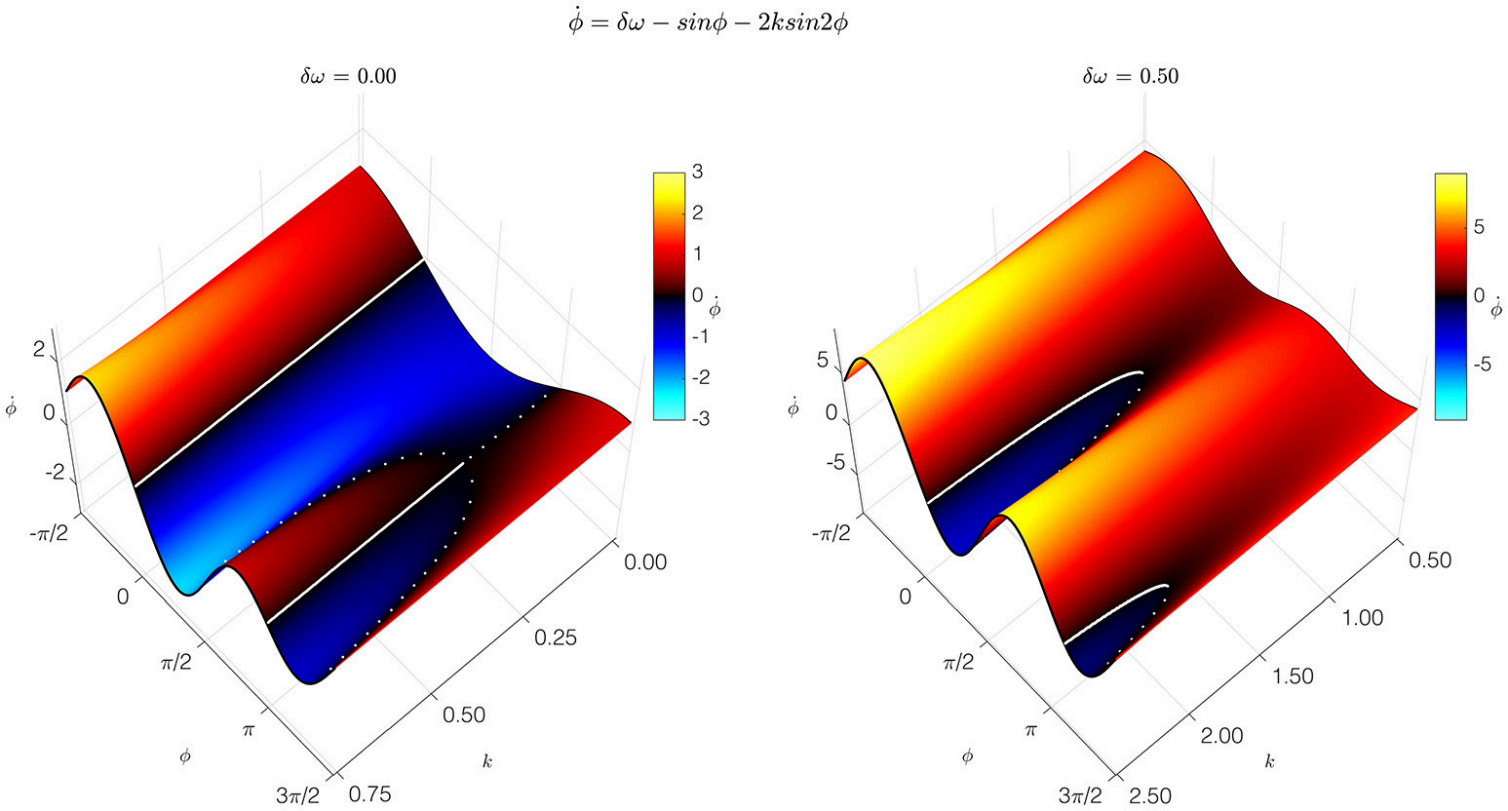
**Coordination dynamics of a coupled dynamical system**. The coordination dynamics is represented by the relation between the relative phase of the coupled components Φ, and its first time derivative $\dot{\phi }$ (vertical axis). It is captured by the extended HKB model, which generated these graphs. Thick solid and broken lines correspond to attractive and repelling fixed points of the dynamics. At the left, the symmetric condition is shown with parameter δω = 0.00. At the right is the broken symmetry condition with parameter δω = 0.50. Slices through the surface in the broken-symmetry case show different dynamics, depending on the control parameter, *k*. For high values of *k*, representing a low frequency (long period) of oscillation, two stable fixed points near Φ = 0° and Φ = 180°, and two unstable fixed points, appear. For intermediate values of *k*, one stable fixed point disappears in a saddle-node bifurcation. For low values of *k*, the remaining stable fixed point disappears the same way. Metastable, intermittent dynamics is observed for low values of *k*: although there are no longer any fixed points, there is still attraction to the remnants of the previously stable states (Modified figure reproduced with permission from Kelso, 1994).

Metastability provides a dynamical explanation of “relative coordination,” and is proposed here to underlie the dynamics of cortical function (see also Kelso, 2001, 2008). Relative coordination allows competition between the tendency of system components to maintain their unique spatiotemporal properties (autonomy) and the opposing tendency to cooperate to produce a unified functioning system (Kelso, 1995).

One way that relative coordination has been studied is by research on “intermittent dynamics” (Kelso and de Guzman, 1991), in which the changing strength of relative coordination in a system is tracked over time. The degree of coordination often remains weak for a long time, only to then suddenly become stronger. The system's intermittent relative phase distribution contains all possible phase values, but these values are concentrated around specific (preferred) phase relations.

The event-related local field potential (LFP) reflects the cortical activity most relevant to interareal cortical relative coordination. The LFP signal derives from extracellular field current flow generated by a neuronal group's synchronous dendritic activity in a cortical tissue volume (Pfurtscheller, 1992; Lopes da Silva, 2013). The LFP does not preserve specific contributions of the group's individual neurons, but it does reveal their common activity, important for understanding the cortical area interactions. Evidence suggests that axonal pulse transmission between areas is organized at the neuronal group level, not at that of the single neuron (Edelman, 1978; Bressler, 2015). The LFP itself is not transmitted between areas, but rather reflects the interareal coordination brought about by pulse activity. Generated by postsynaptic dendritic activity in a neuronal group, the LFP reflects the summation of synaptic inputs received by the dendrites of the neurons in the group, and the summed axonal pulses of the group. The LFP thus reflects the influence that one cortical area exerts on one another. The extracranially recorded EEG and MEG signals are directly related to the intra-cortical LFP signal, and cortical LFP oscillations are directly related to a variety of different cognitive processes (Cavanagh and Frank, 2014; Voytek and Knight, 2015).

The extended HKB model is informative about the dynamic behavior of interacting cortical areas. It suggests that the relative phase of LFPs from different cortical sites can be treated as an order parameter. It seems plausible that the cortex operates under the broken symmetry conditions described above because different cortical areas have characteristic spatiotemporal properties such as oscillation frequency (Hutcheon and Yarom, 2000; Kelso, 2001, 2008). Transmission delays between cortical areas accentuate broken symmetry, as do other interareal influences related to cognitive factors such as intention, learning, memory, and the influence of the environment (Kelso, 1995).

Monkey studies demonstrate that relative coordination is a hallmark of interareal relations in the cortex. During task performance, LFP recordings are made from chronic or semi-chronic microelectrodes in the cerebral cortex (Bressler et al., 1993; Brovelli et al., 2004; Salazar et al., 2012). To track the evolution of task processing, parametric models are derived from the LFPs with high temporal precision, and spectral coherence is computed from the models. Spectral coherence measures LFP relative phase consistency (or phase synchronization), it serves to index interareal relative coordination. Coordination dynamics encompasses interareal synchronization. Specifically, interareal cortical coherence undergoes rapid transitions between low and high values, reflecting partial synchronization of cortical sites, without locking in global synchronization.

The partial interareal phase synchronization (partially consistent relative phase relation) during task processing indicates cortical involvement in neurocognitive function. Salazar et al. (2012) discovered a consistent relative phase relation between lateral prefrontal and posterior parietal cortical areas during the delay period of a delayed match-to-sample task that was specific to the content of working memory. Brovelli et al. (2004) found that elevated coherence is supported by both feedforward and feedback causal influences between cortical areas, and that coordination dynamics can vary considerably across a cortical network. Site pairs from different cortical areas have different coordination dynamics, and contribute differentially to ongoing cognitive function. Many site pairs do not become coordinated in the task while others do, underscoring the selective nature of cortical coordination dynamics.

Complex cognitive function typically involves the participation of areas distributed across the cortex (Goldman-Rakic et al., 1992; Fuster, 1997; Mesulam, 1998; McIntosh, 1999). Our proposal is simply that large-scale relative coordination is an emergent property that crucially contributes to cognitive function by metastably integrating and segregating the activities of distributed cortical areas. The integration of cortical areas depends on their anatomical interconnection by high-velocity cortico-cortical and subcortical pathways (LaBerge, 1990; Llinas et al., 1991; Felleman and Van Essen, 1991; LaBerge et al., 1992; Phillips et al., 2016). The anatomical basis for large-scale cortical segregation derives from the fact that cortical areas are preferentially sequestered as a part of functional systems (Van Essen and DeYoe, 1995). Given the high level of interareal cortical connectivity, we envision that cortical areas are able to (re-)arrange themselves quickly in a large variety of coordinated configurations. Cortico-cortical connectivity suggests that cortical area activity may become coordinated with activity in other connected areas. The cortex may thus organize itself in any configuration within an enormous space of possible configurations.

Consistent coordination of a set of cortical areas in cognitive processing means that those areas comprise a “coordinated network” or coordinative structure. Such coordinated networks are predicted to exhibit high LFP coherence among their component areas. However, not all areas will be coherent at exactly the same frequency or relative phase value. Evidence from a number of different functional systems exists for the participation of cortical areas in coordinated networks. Coordination is observed as coherence, i.e., relative phase synchronization among network sites, with relative phase varying across the network (Brovelli et al., 2004; Salazar et al., 2012). The observation of consistent relative phase relations between cortical areas affords a stronger interpretation of their functional significance, indexed in terms of temporally specific coordination states, than does the observation of areal co-activation by (temporally diffuse) imaging techniques. The large-scale cortical network that is relatively coordinated corresponds to the “functional cluster” (Tononi et al., 1998; Zemanova et al., 2006). Similar dynamic entities have also been observed by task-related functional connectivity analysis of human fMRI BOLD data (Di et al., 2015).

The cortex has been observed to undergo transitions among metastable coordination states when a subject switches from one processing stage to another (Freeman, 2006) or from one mode of behavior to another (Fuchs et al., 1992; Kelso et al., 1992). In the former, the system is proposed to remain in a coordination state for only a fraction of a second, with transitions occuring even more rapidly. We further propose that during transitions the cortical system rapidly breaks functional couplings within one set of areas and establishes new couplings within another set. This flexibility, manifest as relative coordination and underpinned by metastable coordination dynamics, allows the same area to engage in different functions at different stages of processing. The underlying dynamics also permits the system as a whole to switch rapidly between different functions through the reorganization of component areas into different coordinated networks. Nonparticipating areas supporting one coordination state may be recruited into, or selectively engaged in a coordinated network during the transition to another coordination state. Each recruited area affords the possibility of further transitions to new states because it can engage or disengage other areas with which it is connected (Kelso, 1994).

Coordination dynamics also has implications for computation in the cortex. A large number of competing constraints act on a cortical area, and these constraints must be rapidly resolved. Several authors suggest that the cortex satisfies constraints by a relaxation process in which it settles into a globally consistent state that satisfies the multiple constraints on its interacting component areas (Goldman-Rakic et al., 1992; Duncan et al., 1997; Mesulam, 1998; Duncan, 2013; Buckholtz et al., 2015). The network units in artificial neural networks have access to each other's responses and adjust their own responses accordingly. Relaxation processes allow the network to converge to a global result simply by way of local interactions (Churchland and Sejnowski, 1994). Most current artificial networks, however, become trapped in a stable state (fixed point or limit cycle) as a result. The cortex, we postulate, avoids becoming trapped in a stable state by the flexibility of its coordination state: cortical areas reconcile their competing constraints by changing their relative coordination. Relative coordination should be seen as a *tendency* for cortical areas to become coordinated without them becoming fully coordinated in a fixed phase relation. Relative coordination thus prevents the cortex from becoming locked in stable coordination states (Kelso, 1992, 1995).

A distinct advantage of relative coordination is that it creates context for computations in a large-scale network's coordinated areas by way of coordinated network interactions (Phillips and Singer, 1997; Bressler and McIntosh, 2007; Mante et al., 2013; Coen-Cagli et al., 2015). The local creation of computational context thereby dynamically bestows adaptability upon the cortical mechanisms of cognition. Visual cortical neurons, for example, are modulated by many contextual factors (Moran and Desimone, 1985; Phillips and Singer, 1997; Coen-Cagli et al., 2015), possibly enabling the formation of novel groupings in visual perception (Bressler, 1996; Watt and Phillips, 2000; Piëch et al., 2013; Chen et al., 2014). The modulation of local pattern formation by re-entrant processing in a coordinated large-scale network means that network neurons can rapidly adapt to a host of computational contingencies, not just in vision but in all cognitive functions. And, as large-scale coordinated networks dynamically evolve in cognition, re-entrant interactions allow neuronal adaptation to dynamically evolve as task contingencies change.

In computational terms, states of the cortical system are often considered as “solutions” to the problem of adapting to whole-brain processing requirements (Zucker, 2012). On the other hand, if the cortical system operates, as we propose here, in the metastable regime of its dynamics, then, strictly speaking, the “solutions” do not take the form of asymptotically stable states. Rather, they are states of relative coordination. This means that the “solution” is able to change from moment to moment, adaptively evolving with the ongoing reorganization of coordinated networks. In this process, those areas that can resolve the constraints imposed on them, and thus manifest cognitively consistent spatial activity patterns, become instantaneously engaged in a coordinated network, while those areas that cannot are temporarily excluded from the network. This process of adaptive pattern constraint may underlie the formation of coherent movements and percepts as mutual constraint satisfaction powerfully determines the behavior of the entire cortical system.

According to the present proposal, the cerebral cortex critically contributes integration to cognition by combining and reconciling inputs to multiple areas from a multitude of sources. It also contributes segregation by allocating a diversity of tasks to different areas. By rapidly balancing these integrative and segregative functions, the cortex thereby maintains currency with the environment. We argue that metastability, emerging in large-scale coordinated cortical networks, allows network areas to interact while maintaining a degree of independence. By means of metastable coordination dynamics, relative coordination gives cognition the capacity for rapid and fluid change, without the coordinated network ever needing to relax into a stable state.

Such change is highly adaptable. The transient, conjoint coordination of groups of distributed cortical areas comprising large-scale networks can allow individual local-area spatial activity distributions to converge to cognitively consistent patterns, thereby satisfying global computational demands. This computational strategy is extremely flexible, allowing each participating local area to act as a unique source of input for the large-scale network, and different combinations of areas to compute together in real time. The context for computation in each area of the large-scale network is dynamically created by virtue of the combined constraints imposed on it. The result is an enormous computational advantage for perceptual (Phillips and Singer, 1997) and motor (Wise et al., 1997) operations (see also Habenschuss et al., 2013). Through the relative coordination of cortical areas, governed by metastable coordination dynamics, these processes are effectively combined with others into coherent, global functions that give unity to cognition.

## Author contributions

All authors listed, have made substantial, direct and intellectual contribution to the work, and approved it for publication.

### Conflict of interest statement

The authors declare that the research was conducted in the absence of any commercial or financial relationships that could be construed as a potential conflict of interest.

## References

[B1] Bhowmik D. Shanahan M. (2013). Metastability and inter-band frequency modulation in networks of oscillating spiking neuron populations. PLoS ONE 8:e62234. 10.1371/journal.pone.0062234 23614040 PMC3628585

[B2] Braun J. Mattia M. (2010). Attractors and noise: twin drivers of decisions and multistability. Neuroimage 52, 740–751. 10.1016/j.neuroimage.2009.12.126 20083212

[B3] Breakspear M. (2001). Perception of odors by a nonlinear model of the olfactory bulb. Int. J. Neural Syst. 11, 101–124. 10.1142/S0129065701000564 14632166

[B4] Bressler S. L. (1987a). Relation of olfactory bulb and cortex. I. Spatial variation of bulbocortical interdependence. Brain Res. 409, 285–293. 3580877 10.1016/0006-8993(87)90713-x

[B5] Bressler S. L. (1987b). Relation of olfactory bulb and cortex. II. Model for driving of cortex by bulb. Brain Res. 409, 294–301. 3034379 10.1016/0006-8993(87)90714-1

[B6] Bressler S. L. (1995). Large-scale cortical networks and cognition. Brain Res. Brain Res. Rev. 20, 288–304. 7550362 10.1016/0165-0173(94)00016-i

[B7] Bressler S. L. (1996). Interareal synchronization in the visual cortex. Behav. Brain Res. 76, 37–49. 8734042 10.1016/0166-4328(95)00187-5

[B8] Bressler S. L. (2008). Neurocognitive networks. Scholarpedia 3:1567. 10.3389/fpsyg.2012.00571 23267340 PMC3527735

[B9] Bressler S. L. (2015). Chapter 12: Interareal neocortical actions by neuronal populations, in Cognitive Phase Transitions in the Cerebral Cortex – Enhancing the Neuron Doctrine by Modeling Neural Fields, eds Kozma R. Freeman W. J. (Cham: Springer International), 127–134.

[B10] Bressler S. L. Kelso J. A. S. (2001). Cortical coordination dynamics and cognition. Trends Cogn. Sci. 5, 26–36. 10.1016/S1364-6613(00)01564-3 11164733

[B11] Bressler S. L. McIntosh A. R. (2007). The role of neural context in large-scale neurocognitive network operations, in Handbook of Brain Connectivity, eds Jirsa V. K. McIntosh A. R. (Berlin; Heidelberg: Springer Complexity), 403–419.

[B12] Bressler S. L. Coppola R. Nakamura R. (1993). Episodic multiregional cortical coherence at multiple frequencies during visual task performance. Nature 366, 153–156. 10.1038/366153a0 8232553

[B13] Brovelli A. Ding M. Ledberg A. Chen Y. Nakamura R. Bressler S. L. (2004). Beta oscillations in a large-scale sensorimotor cortical network: directional influences revealed by Granger causality. Proc. Natl. Acad. Sci. U.S.A. 101, 9849–9854. 10.1073/pnas.0308538101 15210971 PMC470781

[B14] Buckholtz J. W. Martin J. W. Treadway M. T. Jan K. Zald D. H. Jones O.. (2015). From blame to punishment: disrupting prefrontal cortex activity reveals norm enforcement mechanisms. Neuron 87, 1369–1380. 10.1016/j.neuron.2015.08.023 26386518 PMC5488876

[B15] Bullmore E. Sporns O. (2009). Complex brain networks: graph theoretical analysis of structural and functional systems. Nat. Rev. Neurosci. 10, 186–198. 10.1038/nrn2575 19190637

[B16] Cavanagh J. F. Frank M. J. (2014). Frontal theta as a mechanism for cognitive control. Trends Cogn. Sci. 18, 414–421. 10.1016/j.tics.2014.04.012 24835663 PMC4112145

[B17] Chen M. Yan Y. Gong X. Gilbert C. D. Liang H. Li W.. (2014). Incremental integration of global contours through interplay between visual cortical areas. Neuron 82, 682–694. 10.1016/j.neuron.2014.03.023 24811385

[B18] Churchland P. S. Sejnowski T. J. (1994). The Computational Brain. Cambridge, MA: MIT Press.

[B19] Coen-Cagli R. Kohn A. Schwartz O. (2015). Flexible gating of contextual influences in natural vision. Nat. Neurosci. 18, 1648–1655. 10.1038/nn.4128 26436902 PMC4624479

[B20] Deco G. Jirsa V. K. McIntosh A. R. (2011). Emerging concepts for the dynamical organization of resting-state activity in the brain. Nat. Rev. Neurosci. 12, 43–56. 10.1038/nrn2961 21170073

[B21] Di X. Fu Z. Chan S. C. Hung Y. S. Biswal B. B. Zhang Z. (2015). Task-related functional connectivity dynamics in a block-designed visual experiment. Front. Hum. Neurosci. 9:543. 10.3389/fnhum.2015.00543 26483660 PMC4588125

[B22] Dumas G. de Guzman G. C. Tognoli E. Kelso J. A. S. (2014). The Human Dynamic Clamp as a paradigm for social interaction. Proc. Natl. Acad. Sci. U.S.A. 111, E3726–E3734. 10.1073/pnas.1407486111 25114256 PMC4156776

[B23] Duncan J. (2013). The structure of cognition: attentional episodes in mind and brain. Neuron 80, 33–50. 10.1016/j.neuron.2013.09.015 24094101 PMC3791406

[B24] Duncan J. Humphreys G. Ward R. (1997). Competitive brain activity in visual attention. Curr. Opin. Neurobiol. 7, 255–261. 9142748 10.1016/s0959-4388(97)80014-1

[B25] Edelman G. M. (1978). Group selection and phasic reentrant signaling: a theory of higher brain function, in The Mindful Brain, eds Edelman G. M. Mountcastle V. B. (Cambridge, MA: MIT Press), 55–100.

[B26] Felleman D. Van Essen D. (1991). Distributed hierarchical processing in the primate cerebral cortex. Cereb. Cortex 1, 1–47. 10.1093/cercor/1.1.1 1822724

[B27] Freeman W. J. (2006). A cinematographic hypothesis of cortical dynamics in perception. Int. J. Psychophysiol. 60, 149–161. 10.1016/j.ijpsycho.2005.12.009 16513196

[B28] Fries P. (2005). A mechanism for cognitive dynamics: neuronal communication through neuronal coherence. Trends Cogn. Sci. 9, 474–480. 10.1016/j.tics.2005.08.011 16150631

[B29] Friston K. J. (1997). Transients, metastability, and neuronal dynamics. Neuroimage 5, 164–171. 10.1006/nimg.1997.0259 9345546

[B30] Friston K. Breakspear M. Deco G. (2012). Perception and self-organized instability. Front. Comput. Neurosci. 6:44. 10.3389/fncom.2012.00044 22783185 PMC3390798

[B31] Fuchs A. Kelso J. A. S. Haken H. (1992). Phase transitions in the human brain: spatial mode dynamics. Int. J. Bifurcat. Chaos 2, 917–939.

[B32] Fuchs A. Mayville J. M. Cheyne D. Weinberg H. Deecke L. Kelso J. A. S. (2000). Spatiotemporal analysis of neuromagnetic events underlying the emergence of coordinative instabilities. Neuroimage 12, 71–84. 10.1006/nimg.2000.0589 10875904

[B33] Fuster J. M. (1997). Network memory. Trends Neurosci. 20, 451–459. 10.1017/S1355617714000800 9347612

[B34] Goldman-Rakic P. S. Chafee M. Friedman H. (1992). Allocation of function in distributed circuits, in Brain Mechanisms of Perception and Memory: From Neuron to Behavior, eds Ono T. Squire L. R. Raichle M. E. Perrett D. I. Fukuda T. (Oxford: Oxford University Press), 445–456.

[B35] Habenschuss S. Jonke Z. Maass W. (2013). Stochastic computations in cortical microcircuit models. PLoS Comput. Biol. 9:e1003311. 10.1371/journal.pcbi.1003311 24244126 PMC3828141

[B36] Haken H. (1996). Principles of Brain Functioning. Berlin; Heidelberg: Springer.

[B37] Haken H. Kelso J. A. S. Bunz H. (1985). A theoretical model of phase transitions in human hand movements. Biol. Cybern. 51, 347–356. 10.1007/BF00336922 3978150

[B38] Hutcheon B. Yarom Y. (2000). Resonance, oscillation and the intrinsic frequency preference of neurons. Trends Neurosci. 23, 216–222. 10.1016/S0166-2236(00)01547-2 10782127

[B39] Jantzen K. J. Steinberg F. L. Kelso J. A. S. (2009). Coordination dynamics of large-scale neural circuitry underlying sensorimotor behavior. J. Cogn. Neurosci. 21, 2420–2433. 10.1162/jocn.2008.21182 19199411

[B40] Kelso J. A. S. (1992). Coordination dynamics of human brain and behavior. Springer Proc. Physics 69, 223–234.

[B41] Kelso J. A. S. (1994). The informational character of self-organized coordination dynamics. Hum. Mov. Sci. 13, 393–413.

[B42] Kelso J. A. S. (1995). Dynamic Patterns: The Self-Organization of Brain and Behavior. Cambridge, MA: MIT Press

[B43] Kelso J. A. S. (2000). Principles of dynamic pattern formation and change for a science of human behavior, in Developmental Science and the Holistic Approach, eds Bergman L. R. Cairns R. B. Nilsson L-G. Nystedt L. (Hillsdale, NJ: Erlbaum), 63–83.

[B44] Kelso J. A. S. (2008). An essay on understanding the mind. Ecol. Psychol. 20, 180–208. 10.1080/10407410801949297 19865611 PMC2768408

[B45] Kelso J. A. S. (2012). Multistability and metastability: understanding dynamic coordination in the brain. Philos. Trans. R. Soc. Lond. B Biol. Sci. 367, 906–918. 10.1098/rstb.2011.0351 22371613 PMC3282307

[B46] Kelso J. A. S. (2014). The dynamic brain in action: Coordinative structures, criticality and coordination dynamics, in Criticality in Neural Systems, eds Plenz D. Niebur E. (Mannheim: John Wiley & Sons), 67–106.

[B47] Kelso J. A. S. de Guzman G. C. (1991). An intermittency mechanism for coherent and flexible brain and behavioral function, in Tutorials in Motor Neuroscience, eds Requin J. Stelmach G. E. (Amsterdam), 305–310.

[B48] Kelso J. A. S. Haken H. (1995). ew laws to be expected in the organism: synergetics of brain and behavior, in What is Life? The Next 50 Years, eds Murphy M. O'Neill L. (Cambridge: Cambridge University Press), 137–160.

[B49] Kelso J. A. S. Bressler S. L. Buchanan S. de Guzman G. C. Ding M. Fuchs A. (1992). A phase transition in human brain and behavior. Phys. Lett. A 169, 134–144.

[B50] Kelso J. A. S. de Guzman G. C. Reveley C. Tognoli E. (2009). Virtual Partner Interaction (VPI): exploring novel behaviors via coordination dynamics. PLoS ONE 4:e5749. 10.1371/journal.pone.0005749 19492044 PMC2685001

[B51] Kelso J. A. S. DelColle J. D. Schoner G. (1990). Action-perception as a pattern formation process, in Attention and Performance XIII, ed Jeannerod M. (Hillsdale, NJ: Erlbaum), 139–169.

[B52] Kelso J. A. S. Dumas G. Tognoli E. (2013). Outline of a general theory of behavior and brain coordination. Neural Netw. 37, 120–131. 10.1016/j.neunet.2012.09.003 23084845 PMC3914303

[B53] Kelso J. A. S. Tognoli E. Dumas G. (2014). Coordination dynamics: bidirectional coupling between humans, machines and brains. IEEE Intl. Conf. Syst. Man Cybern, 2269–2272. 10.1109/smc.2014.6974258

[B54] Kelso J. A. S. (2001). Metastable coordination dynamics of brain and behavior. Brain Neural Netw. 8, 125–130. 10.3902/jnns.8.125

[B55] LaBerge D. (1990). Thalamic and cortical mechanisms of attention suggested by recent positron tomographic experiments. J. Cogn. Neurosci. 2, 358–372. 23964760 10.1162/jocn.1990.2.4.358

[B56] LaBerge D. Carter M. Brown V. (1992). A network simulation of thalamic circuit operations in selective attention. Neural Comput. 4, 318–331. 10.1162/neco.1992.4.3.318

[B57] Llinas R. Grace A. A. Yarom Y. (1991). *In vitro* neurons in mammalian cortical layer 4 exhibit intrinsic oscillatory activity in the 10- to 50-Hz frequency range. Proc. Natl. Acad. Sci. U.S.A. 88, 897–901. 1992481 10.1073/pnas.88.3.897 PMC50921

[B58] Lopes da Silva F. (2013). EEG and MEG: relevance to neuroscience. Neuron 80, 1112–1128. 10.1016/j.neuron.2013.10.017 24314724

[B59] Luria A. R. (1980). Higher Cortical Functions in Man. Dordrecht: Kluwer Academic Publishers.

[B60] Mante V. Sussillo D. Shenoy K. V. Newsome W. T. (2013). Context-dependent computations by recurrent dynamics in prefrontal cortex. Nature 503, 78–84. 10.1038/nature12742 24201281 PMC4121670

[B61] Mayville J. M. Bressler S. L. Fuchs A. Kelso J. A. S. (1999). Spatiotemporal reorganization of electrical activity in the human brain associated with a timing transition in rhythmic auditory-motor coordination. Exp. Brain Res. 127, 371–381. 10.1007/s002210050805 10480272

[B62] McIntosh A. R. (1999). Mapping cognition to the brain through neural interactions. Memory 7, 523–548. 10.1080/096582199387733 10659085

[B63] Meehan T. P. Bressler S. L. (2012). Neurocognitive networks: findings, model, and theory. Neurosci. Biobehav. Rev. 36, 2232–2247. 10.1016/j.neubiorev.2012.08.002 22921284

[B64] Mesulam M. M. (1998). From sensation to cognition. Brain 121, 1013–1052. 10.1093/brain/121.6.1013 9648540

[B65] Meyer-Lindenberg A. Ziemann U. Hajak G. Cohen L. Berman K. F. (2002). Transitions between dynamical states of differing stability in the human brain. Proc. Natl. Acad. Sci. U.S.A. 99, 10948–10953. 10.1073/pnas.162114799 12151599 PMC123190

[B66] Moran J. Desimone R. (1985). Selective attention gates visual processing in extrastriate cortex. Nature 229, 782–784. 10.1126/science.4023713 4023713

[B67] Pfurtscheller G. (1992). Event-related synchronization (ERS): an electrophysiological correlate of cortical areas at rest. Electroenceph. Clin. Neurophysiol. 83, 62–69. 1376667 10.1016/0013-4694(92)90133-3

[B68] Phillips J. M. Kambi N. A. Saalmann Y. B. (2016). A subcortical pathway for rapid, goal-driven, attentional filtering. Trends Neurosci. 39, 49–51. 10.1016/j.tins.2015.12.003 26743499

[B69] Phillips W. A. Singer W. (1997). In search of common foundations for cortical computation. Behav. Brain Sci. 20, 657–722. 10.1017/S0140525X9700160X 10097008

[B70] Piëch V. Li W. Reeke G. N. Gilbert C. D. (2013). Network model of top-down influences on local gain and contextual interactions in visual cortex. Proc. Natl. Acad. Sci. U.S.A. 110, E4108–E4117. 10.1073/pnas.1317019110 24101495 PMC3808648

[B71] Rabinovich M. I. Afraimovich V. S. Bick C. Varona P. (2012). Information flow dynamics in the brain. Phys. Life Rev. 9, 51–73. 10.1016/j.plrev.2011.12.007 22119154

[B72] Razi A. Friston K. (2016). The connected brain: causality, models and intrinsic dynamics. IEEE Signal Process. 33, 14–35. 10.1109/MSP.2015.2482121

[B73] Salazar R. F. Dotson N. M. Bressler S. L. Gray C. M. (2012). Content-specific fronto-parietal synchronization during visual working memory. Science 338, 1097–1100. 10.1126/science.1224000 23118014 PMC4038369

[B74] Schöner G. Kelso J. A. S. (1988). Dynamic pattern generation in behavioral and neural systems. Science 239, 1513–1520. 10.1126/science.3281253 3281253

[B75] Schöner G. Haken H. Kelso J. A. S. (1986). A stochastic theory of phase transitions in human hand movement. Biol. Cybern. 53, 247–257. 10.1007/BF00336995 3955100

[B76] Shanahan M. (2010). Multistable chimera states in community-structured oscillator networks. Chaos 20, 013108. 10.1063/1.3305451 20370263

[B77] Shew W. L. Yang H. Yu S. Roy R. Plenz D. (2011). Information capacity and transmission are maximized in balanced cortical networks with neuronal avalanches. J. Neurosci. 31, 55–63. 10.1523/JNEUROSCI.4637-10.2011 21209189 PMC3082868

[B78] Singer W. (1994). Coherence as an organizing principle of cortical functions. Int. Rev. Neurobiol. 37, 153–183. 7883477 10.1016/s0074-7742(08)60245-7

[B79] Solé R. V. Alonso D. McKane A. (2002). Self-organized instability in complex ecosystems. Philos. Trans. R. Soc. Lond. B. Biol. Sci. 357, 667–671. 10.1098/rstb.2001.0992 12079528 PMC1692980

[B80] Tognoli E. Kelso J. A. S. (2009). Brain coordination dynamics; true and false faces of phase synchrony and metastability. Prog. Neurobiol. 87, 31–40. 10.1016/j.pneurobio.2008.09.014 18938209 PMC3020160

[B81] Tognoli E. Kelso J. A. S. (2014). The metastable brain. Neuron 81, 35–48. 10.1016/j.neuron.2013.12.022 24411730 PMC3997258

[B82] Tononi G. Edelman G. M. Sporns O. (1998). Complexity and coherency: integrating information in the brain. Trends Cogn. Sci. 2, 474–484. 21227298 10.1016/s1364-6613(98)01259-5

[B83] Tononi G. Sporns O. Edelman G. M. (1994). A measure for brain complexity: Relating functional segregation and integration in the nervous system. Proc. Natl. Acad. Sci. U. S. A. 91, 5033–5037. 8197179 10.1073/pnas.91.11.5033 PMC43925

[B84] Van Essen D. C. DeYoe E. (1995). Concurrent processing in the primate visual cortex, in The Cognitive Neurosciences, ed Gazzaniga M. S. (Cambridge, MA: MIT Press), 383–400.

[B85] von Holst E. (1939/1973). Relative coordination as a phenomenon as a method of analysis of central nervous function, in The Collected Papers of Erich von Holst, ed Martin R. (Miami, FL: University of Miami Press), 33–135.

[B86] Voytek B. Knight R. T. (2015). Dynamic network communication as a unifying neural basis for cognition, development, aging, and disease. Biol. Psychiatry 77, 1089–1097. 10.1016/j.biopsych.2015.04.016 26005114 PMC4443259

[B87] Wallenstein G. V. Kelso J. A. S. Bressler S. L. (1995). Phase transitions in spatiotemporal patterns of brain activity and behavior. Physica D 84, 626–634. 10.1016/0167-2789(95)00056-A

[B88] Watt R. J. Phillips W. A. (2000). The function of dynamic grouping in vision. Trends Cogn. Sci. 4, 447–454. 10.1016/S1364-6613(00)01553-9 11115758

[B89] Wise S. P. Boussaoud D. Johnson P. B. Caminiti R. (1997). Premotor and parietal cortex: corticocortical connectivity and combinatorial computations. Ann. Rev. Neurosci. 20, 25–42. 10.1146/annurev.neuro.20.1.25 9056706

[B90] Zemanova L. Zhou C. Kurths J. (2006). Structural and functional clusters of complex brain networks. Physica D 224, 202–212. 10.1016/j.physd.2006.09.008

[B91] Zucker S. (2012). Local field potentials and border ownership: a conjecture about computation in visual cortex. J. Physiol. Paris 106, 297–315. 10.1016/j.jphysparis.2012.08.001 22940191

